# Granular cooling of ellipsoidal particles in microgravity

**DOI:** 10.1038/s41526-022-00196-6

**Published:** 2022-04-20

**Authors:** Sebastian Pitikaris, Patricia Bartz, Peidong Yu, Samantha Cristoforetti, Matthias Sperl

**Affiliations:** 1 Institut für Materialphysik im Weltraum, Deutsches Zentrum für Luft- und Raumfahrt (DLR), 51170 Köln, Germany; 2 Institut für Theoretische Physik, Universität zu Köln, 50937 Köln, Germany; 3 European Astronaut Centre, 51147 Köln, Germany

**Keywords:** Statistical physics, thermodynamics and nonlinear dynamics, Fluid dynamics

## Abstract

A three-dimensional granular gas of ellipsoids is established by exposing the system to the microgravity environment of the International Space Station. We use two methods to measure the dynamics of the constituent particles and report the long-time development of the granular temperature until no further particle movement is detectable. The resulting cooling behavior can be well described by Haff’s cooling law with time scale *τ*. Different analysis methods show evidence of particle clustering towards the end of the experiment. By using the kinetic theory for ellipsoids we compare the translational energy dissipation of individual collision events with the overall cooling time scale *τ*. The difference from this comparison indicates how energy is distributed in different degrees of freedom including both translation and rotation during the cooling.

## Introduction

The ideal gas equation as the relationship between pressure, volume, and temperature is a well-known law for thermal gases. Although there are many analogies between the statistical physics of thermal and granular systems, granular particles exhibit a unique cooling behavior when left without agitation. This is because the interaction in granular systems happens exclusively over particle contacts. In a granular gas these collisions between particles are inelastic and the system will dissipate energy over time, i.e., it will cool. Haff was the first to look at the cooling of granular flows in 1983^1^. He predicts that the mean velocity of the constituent particles decreases algebraically for dilute systems, i.e., granular gases. This prediction has been tested and confirmed several times^2–6^. Another consequence of the inelastic nature of the particle interactions has been proposed to be the clustering of the particles, as granular gases with large amount of particles can separate into dense and dilute regions^7–10^. Apart from theoretical ideas and numerical simulations, some experiments have also been performed to validate this prediction. Experimentally, granular gases can be established by placing granular particles into weightlessness. Therefore, one either has to counter the gravitational force in the framework of a levitation experiment^11–13^ or by providing a microgravity environment such as in a droptower experiment or a parabolic flight^14–16^. Most of the experiments were conducted in two dimensions. Only recently also three-dimensional systems were investigated^6,13,17,18^, which are expected to show clustering already at rather low-volume fractions^19,20^. Obviously, the driving protocol as well as the detailed environmental parameters to establish weightlessness have a strong influence on the dynamics of the system^17,21^.

**Fig. 1:**
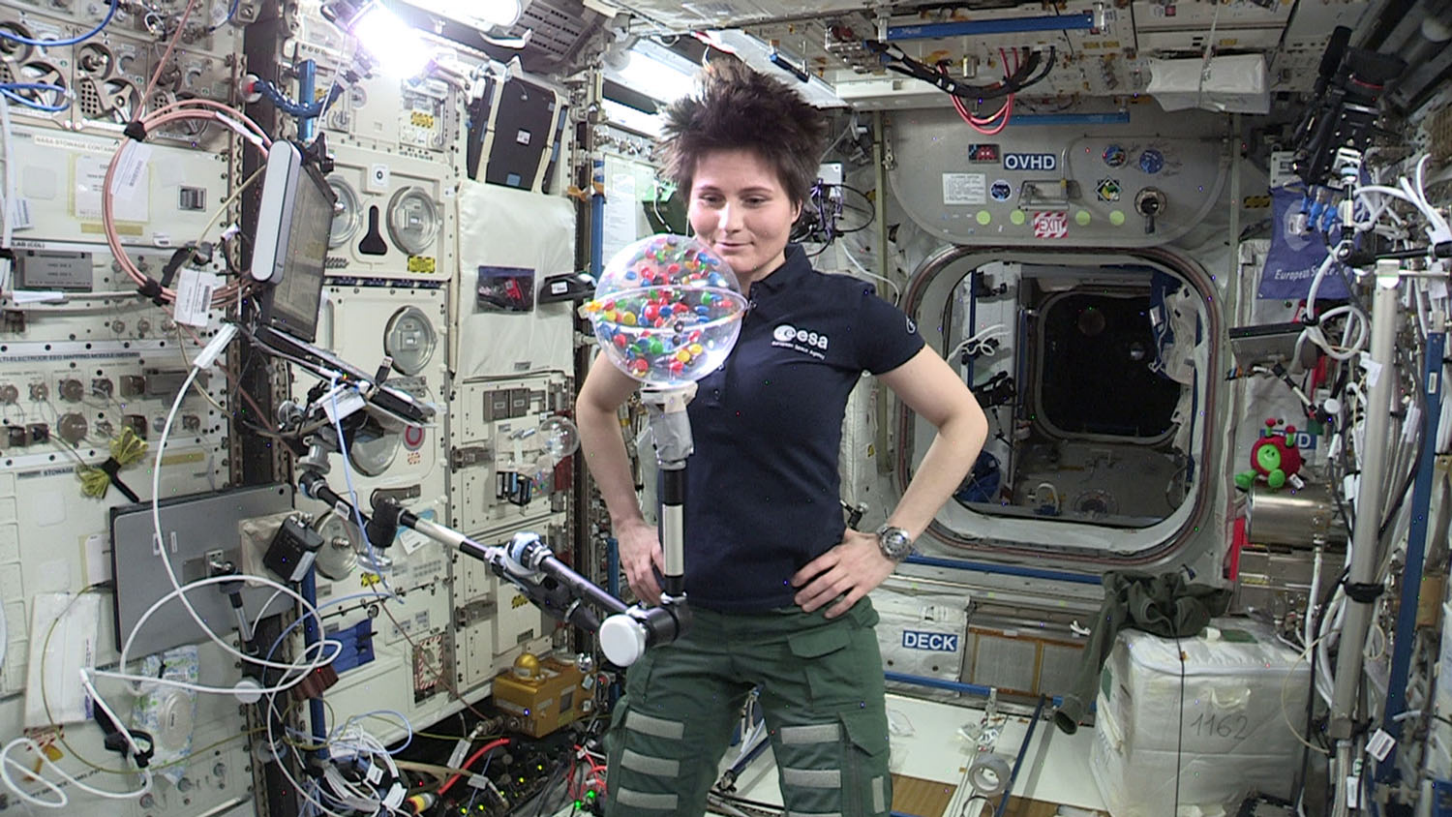
Experiment in ISS. Astronaut Samantha Cristoforetti conducting the experiment on board the ISS during Expedition 42/43.

In this work, we investigate the cooling behavior of a three-dimensional granular gas constituted of ellipsoidal particles. The investigation was conducted as an educational experiment on board the International Space Station (ISS, cf. Fig. 1), which provides a high-quality microgravity environment for long times (~80 s for one experiment). Among the existing experimental works, Harth et al.^18^ investigated similar non-spherical particles in low-gravity conditions and verified the theoretical prediction of the collision rate^22^ during homogeneous cooling. The relatively short low-gravity time (~9 s) limited by the low-gravity platform prevents analysis of long-term cooling behavior. To our knowledge there is no other experimental investigation of non-spherical particle systems under low-gravity before the current one.

Our granular system consists of 96 commercially available Mars Inc. M&M candies, which have an ellipsoidal shape with a short axis of radius *a* ≃ 3.50 mm and two identical long axes of radius *b* ≃ 6.75 mm (i.e., an oblate ellipsoid). The mass of one such ellipsoid *m* is about 0.91 g. The container has a spherical shape and is made of synthetic material and has a diameter of about 150 mm. The packing fraction of our system is thus about 3.6%. The video footage is taken by a Canon XF305 at a frame rate of 29.97 fps. The container is shaken up randomly by hand for several seconds to reach an observable homogeneous spatial distribution of the particles, and then placed onto a holder where the video camera records the dynamics of the cooling particles from above. We adopt two image-processing methods to analyze the videos and obtain the mean translational velocity 〈*v*(*t*)〉 in two dimensions. These results are then qualitatively and quantitatively compared with the cooling theory and help us detect the signatures of clustering.

## Methods

**Fig. 2:**
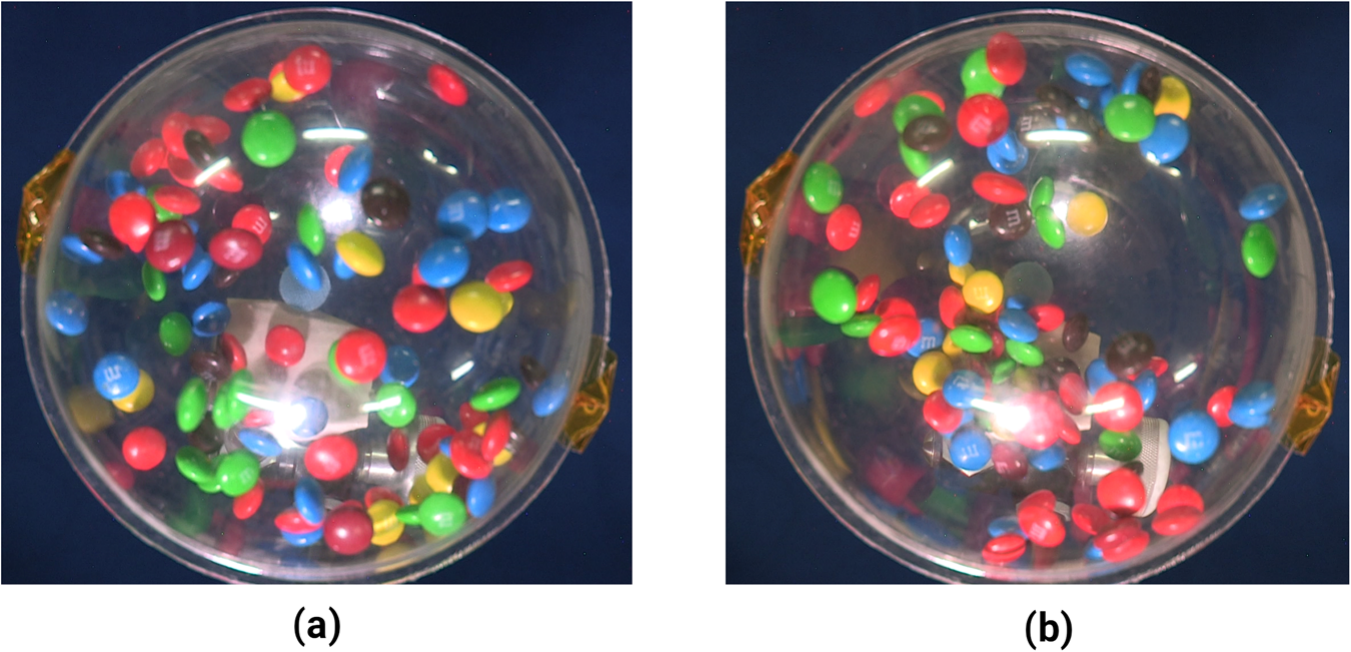
Particle clustering. In four out of the total five experiments we observe first a homogeneous distribution of the particles as shown by **a**, and then a separation of the particles into dense and dilute regions as shown by **b**.

There are in total five experiments performed during the mission. Four of them have problems of minor misplacement and/or unstable positioning of the sample cell under the camera view, which prevent us from applying image processing for quantitative analysis. They are nevertheless useful for our qualitative observation of the clustering behavior as shown in Fig. 2.

### Two image-processing approaches

We implemented two methods to evaluate the cooling dynamics of the one experiment with the best quality. A first approach is based on the optical flow method of Lucas and Kanade^23^. The algorithm tracks so-called features that are unique regions in two successive images determined by high-intensity gradients. We subtract successive images with a distance of Δ = 2, 3, and 8 frames, with the former two parameters yielding very similar results and thus only Δ = 2 presented in the Results section. The resulting difference images provide the advantage of reducing the static reflections on the container walls (e.g., as can been observed in Fig. 2). This rather robust algorithm is easy to implement and gives a good estimate of the mean velocity of the particles if the only changing pixels in the images are caused by particle movement. A similar method was employed for the evaluation in a dense granular system under X-ray radiography^24^. In our evaluation, however, the artifacts such as the reflections can not be completely removed by subtracting images. More importantly, the changing features related with the particle movement themselves are partially caused by particle rotation. The resulting calculation of the translational velocities of the particles thus overestimates the real values.

**Fig. 3:**
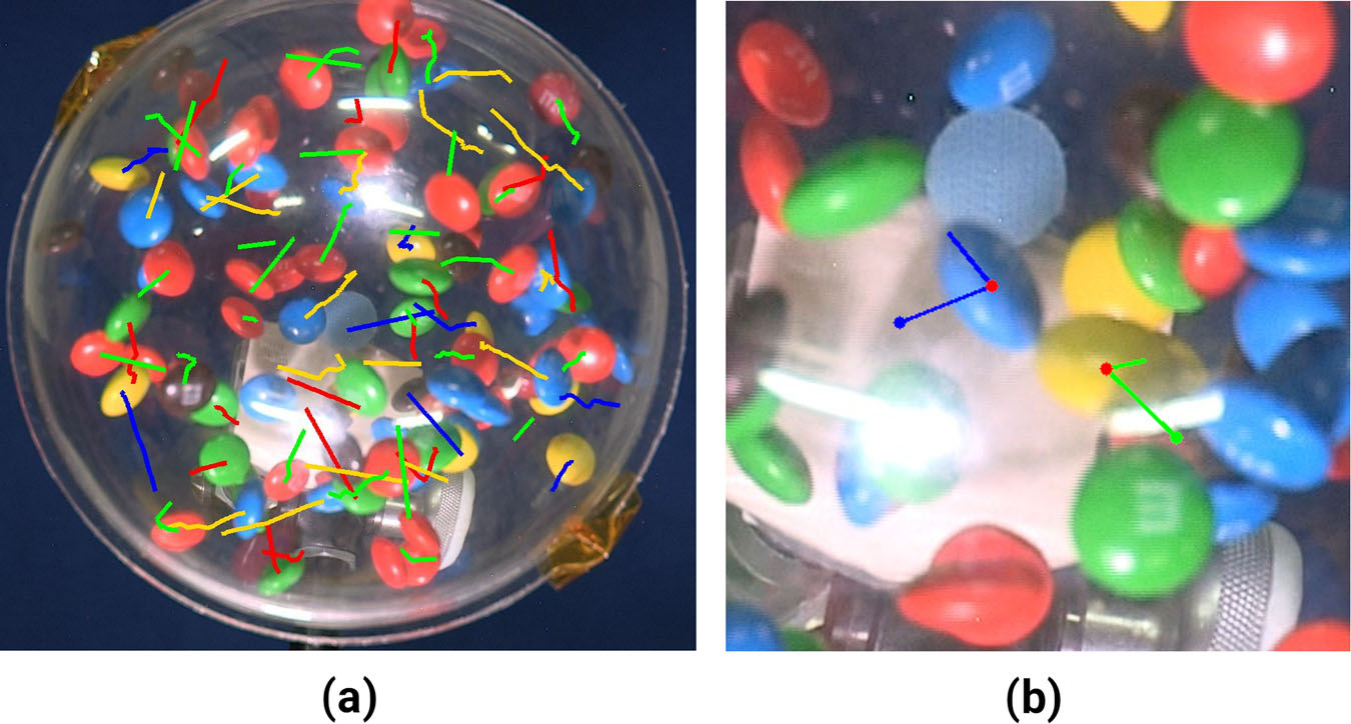
Particl tracking. **a** Proximity-based particle tracking: the ellipsoidal particles are tracked by fitting an ellipse to the individual objects in the image. The trajectories are reconstructed by linking the positions of nearest neighbors in two successive images. If particles get lost in the procedure, their trajectories are interpolated. **b** Collision tracking: a snapshot of one collision event: following the trajectory tracking, collisions fitting with two-dimensional measurement are detected and analyzed. See supplementary videos collision1.avi and collision2.avi for complete events.

In a second approach, we try to reconstruct the trajectory of each particle in the video footage. We miss depth information as we have a two-dimensional projection of a three-dimensional experiment. As a further consequence, we lose track of particles that move behind each other in the projection. We reduced this effect by performing the analysis for the different colors of the particles separately. In each frame the particles are identified by fitting an ellipse to connected objects of the same color. By means of a proximity-based tracking procedure the trajectories can be reconstructed among images (cf. Fig. 3a). Whenever particles get out of sight, their positions are linearly interpolated.

### Collision characterization

During the particle tracking, some collision events between the particles are used for measurement of energy dissipation rate (cf. Fig. 3b). We manually select those events with the motions of both particles before and after the collisions approximately within a plane vertical to the camera view. In other words, we consider in our selected collision events that the changes of the depths of two particles are small compared to their changes of speed in this plane, and the measured translational kinetic energies from the two-dimensional image analysis are close to their three-dimensional values. We measure the translational speeds (*v*_1_, *v*_2_) and $(v^{\prime}_{1} ,v^{\prime}_{2} )$ of the two colliding particles before and after the collision and calculate the translational kinetic energy *T* and $T^{\prime}$ before and after collisions. The ratio

1 $$r=\sqrt{\frac{T^{\prime} }{T}}=\sqrt{\frac{{v^{\prime 2} _{1}}+{v^{\prime 2} _{2}}}{{v}_{1}^{2}+{v}_{2}^{2}}}$$

is used along with the kinetic theory in the previous sections to calculate the cooling time scale. Note that *r* is not the coefficient of restitution *ϵ* that is commonly used in such calculations. Discussions about its usage can be found in the previous section.

As discussed in the previous section, a three-dimensional diagnostics of both the translational and rotational motion of the particles shall be the major goal of our future improvement of the setup.

### Reporting summary

Further information on research design is available in the Nature Research Reporting Summary linked to this article.

## Results

The first image-processing method analyzes the difference between two images close to each other in the time sequence. The optical flow method of Lucas and Kanade^23^ is then used to track the movement of the features from the difference images to estimate the velocity of the particles.

### Comparison between the two methods

The second method is a direct two-dimensional tracking of individual particles and a partially manual reconstruction of the trajectories, which provides the translational velocity. Manual collision event detection also becomes possible with these trajectories, yielding the rate of translational energy dissipation of individual collisions: *r*. The Methods section provides more details of both approaches.

**Fig. 4:**
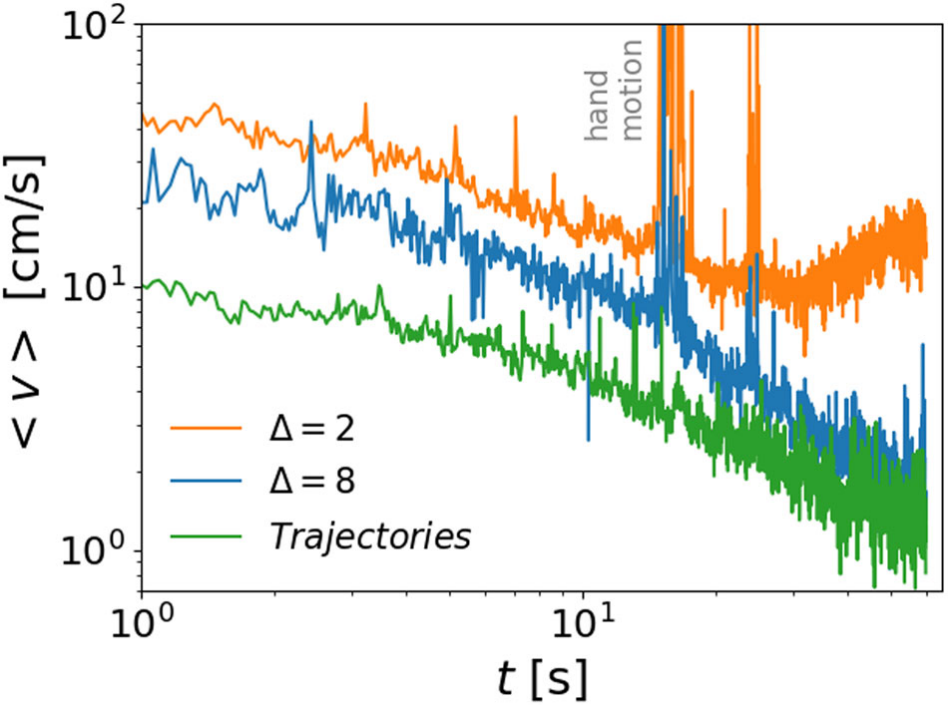
Mean velocities measured by two image-processing methods. Spikes from optical flow results correspond to artifacts from the experimenter’s hand motion reflected on the sample cell, while the rising trend at the end of Δ = 2 curve is due to over-estimation of fast moving particles.

Both methods provide us *v*, the magnitude of the two-dimensional projection of the three-dimensional translational velocities of the particles onto the plane of the camera view. Figure 4 exhibits the development of the average of these velocities 〈*v*(*t*)〉 over time for different analysis methods. For the *n*th and (*n* + Δ)th frames extracted from the experiment video, the parameter Δ is the index difference between two frames to calculate a difference image, which is provided as the source data for the optical flow method. All data shows the general trend of cooling. However, if the index difference is small (Δ = 2), the optical flow analysis shows an increase in the mean velocity at the end of the experiment. This effect vanishes for larger Δ and it is also not visible in the data obtained from particle tracking (trajectories). Similar curves were measured by ref. ^13^, where towards the end of the experiment particles gathered in a cluster and could thus not be tracked anymore. Consequently, only fast moving particles outside the cluster are part of their statistics, which leads to an over-estimation of the system’s kinetic energy in the presence of a cluster. Here, we propose a similar origin: by setting Δ to a low value, slowly moving particles can no longer be detected from the difference of two temporally close images, and can thus not be tracked anymore. In other words, fast particles are in this case over-represented. With higher values of Δ or by tracking particles’ trajectories, this effect disappears. This behavior is only seen in a few measurements as it occurs only in experiments where a phase separation into fast and slow moving particles develops.

### Cooling

**Fig. 5:**
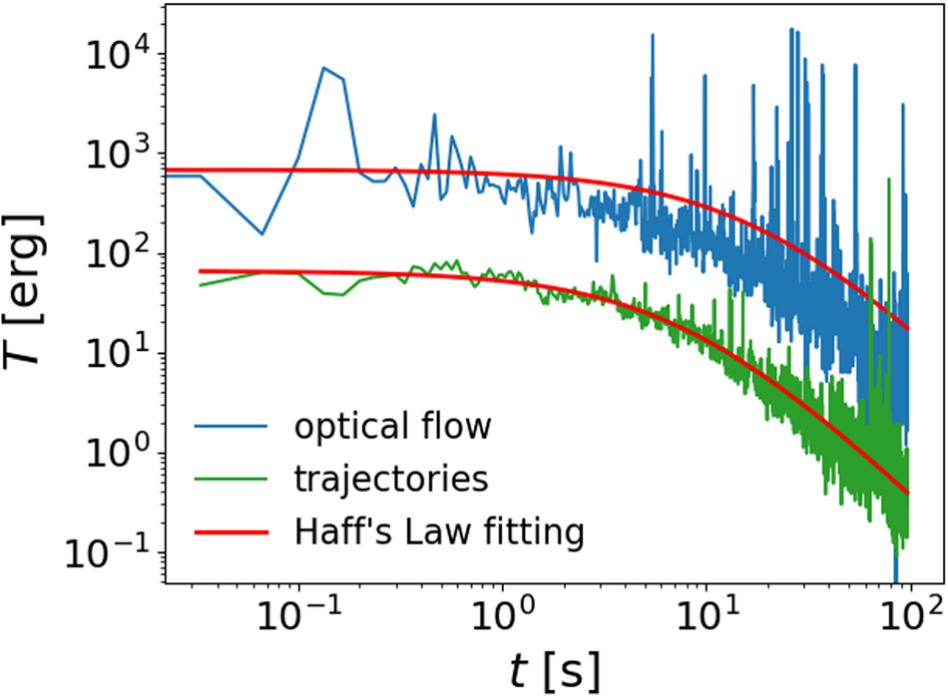
Cooling dynamics. Cooling dynamics of translational motion of the particles.

The cooling dynamics of the system is exhibited in Fig. 5 as the decay of the translational temperature in three dimensions *T* = 2*m*〈*v*〉^2^/*π*. The two curves show the results for the two analysis methods. The measurement from the trajectory tracking is in good agreement with Haff’s law, which is indicated by the fit (solid red line):

2 $$T=\frac{{T}_{0}}{{(1+t/\tau )}^{2}},$$

where *τ* ≃ 8.1 s and *T*_0_ ≃ 6.5 μJ is the fitted Haff time and the initial temperature.

The values obtained by optical flow are on a higher scale in comparison to data obtained from trajectory tracking. This is expected as the optical flow method tends to over-estimate the mean velocity of particles (see Methods section for details). Artifacts in the source images are also tracked as features and result in overall higher values than the true average velocities. This explanation can be also verified by the apparent deviation of Haff’s law fitting of the blue curve when *t* becomes large, since the temporal development of this artifact-induced features do not necessarily follow Haff’s cooling. We therefore use the fitting results of the trajectory tracking data for further discussions.

As described in the Methods section, during the cooling, we follow the particle motions of 86 collision events and measure the ratios of the total translational kinetic energy of the pair of particles before and after the collisions in three dimensions ${r}^{2}=T^{\prime} /T$, resulting in the average value 〈*r*〉 = 0.952. To estimate the overall cooling time scale from this collision property, we refer to the kinetic theory for ellipsoids proposed by^22,25,26^, which gives the collision rate:

3 $${{\Gamma }}=4{\left(\frac{\pi T}{m}\right)}^{\frac{1}{2}}n{g}_{c}{S}_{c}{\langle D\rangle }_{c}.$$

Here, *n* is the number density of particles, *g* is the value of the particle pair distribution function at contact, 4*πS* is the exclusion surface of two contacting ellipsoidal particles, and *D* is a function measuring the energy transfer between rotational and translational degrees of freedom. The subscript “c” on the latter three parameters means to take the average of them over all collision situations. We follow^22,27^ and obtain *g*_c_ = 1.104. We follow^26^ and obtain *S*_c_ ≃ 131 mm^2^ for our oblate particles. As for the transfer function, we follow^22,26^ and perform a numerical integral to obtain 〈*D*〉_*c*_ ≃ 1.218 (see the Supplementary Material for more details of these calculations). Eq. (2) now becomes Γ(*T*) = *C* ⋅ *T*^1/2^, where the prefactor *C* = 0.0712 g^–1/2^mm^−1^.

We then consider that, within a time interval Δ*t*, a total number of Γ(*T*) ⋅ Δ*t* collisions happen with the average loss of translational energy for each collision being (1 − 〈*r*〉^2^) ⋅ *T*. We use this average loss to calcualte the total change Δ*T* = − (1 − 〈*r*〉^2^)Γ(*T*) ⋅ *T*Δ*t* within the time interval Δ*t*, the validity of which is discussed in the Discussion section. Setting the interval infinitesimal, we have:

4 $$\frac{\,{{{\rm{d}}}}T}{\,{{{\rm{d}}}}t}=-(1-{\langle r\rangle }^{2}){{\Gamma }}(T)\cdot T=-(1-{\langle r\rangle }^{2})C\cdot {T}^{\frac{3}{2}},$$

the solution of which is Haff’s law of Eq. (1), with the cooling time scale

5 $${\tau }_{k.t.}=\frac{2}{(1-{\langle r\rangle }^{2}){{\Gamma }}({T}_{0})}.$$

Using *T*_0_ ≃ 6.5 μJ from the data in Fig. 5 and the average value 〈*r*〉 = 0.952, we have *τ*_*k*.*t*._ ≃ 3.8 s, only about half of *τ* = 8.1 s previously measured from the overall cooling curve. In other words, the experiment cools significantly slower than the theoretical prediction.

## Discussion

### Cluster formation

There are experimental studies which report on the formation of clusters in granular gases^11–13^. It should be noted, however, that the preparation of the gas, in particular the agitation as well as the establishment of the weightlessness can have a strong influence on the outcome of the experiment^21^. A clean microgravity environment in the absence of any other interaction force than by collision is difficult to achieve. In this work, we had the benefit of a very clean and enduring microgravity environment. As shown in Fig. 2, we can visually find apparent clustering of the particles at the later stage (>40 s) of most of the experiments, while as shown in the Results section, different cooling behaviors derived by the optical flow method using different Δ also indicate the existence of clustering.

### The cooling time scale and the limitation of *r*

By measuring the decay of the translational temperature and the collision property we were able to quantitatively compare the cooling time scale derived from the experiment with that from the kinetic theory. The difference between the two time scales is in contrast with previous results of three-dimensional works^6,18^, which observed faster cooling in experiment than predicted by kinetic theories. However, this difference is very sensitive to the parameter *r* measured from the collision events.

Note that unlike the coefficient of restitution *ϵ* that is commonly used in kinetic theoretical calculation, *r* only addresses the apparent change of the translational energy. The measurement of *ϵ* from the collision of two ellipsoidal particles requires the tracking of the rotational motion in addition to the translational motion, which can not be reasonably achieved by the current two-dimensional diagnostics. Limiting ourselves only in the translational degree of freedom by using *r*, we are able to disregard the distribution of the energy loss in *n* degrees of freedom when deriving Eq. (3), while when using a proper *ϵ*, an additional prefactor 1/2*n* must be added to the RHS of the equation. The *r* values, however, can be very different from different collision events, and the usage of their overall average value through the whole cooling time period in Eq. (3) indeed needs to be examined.

### Energy partition

**Fig. 6:**
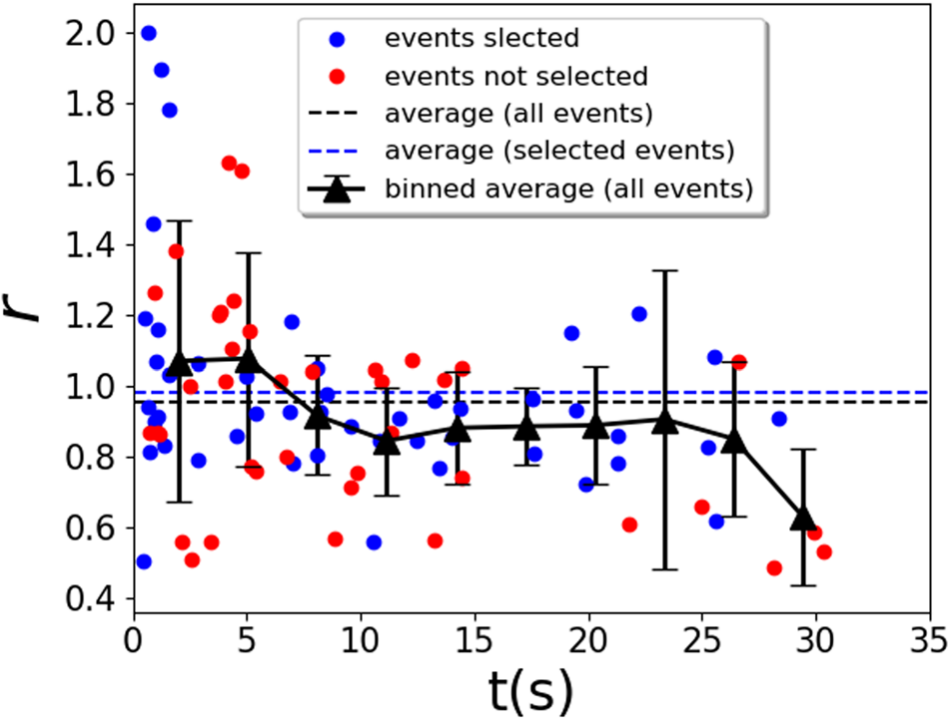
*r* measured from 86 collision events (blue and red circles) during one experiment with the overall average value indicated by the black dashed line. When adopting the criterion of using those collision events with only unambiguously tracked trajectories before and after, 47 blue circles remain and give an average value indicated by the blue dashed line. Black triangles show the average values within every 3 s. Collision events after 30 s into cooling are too difficult to be clearly detected due to the very slow motion of the particles and possible clustering behaviors.

Figure 6 shows the details of *r* measured from 86 collision events at different times *t* after the onset of the cooling. The average value 〈*r*〉 = 0.952 is indicated by the black dashed line. As described in the Methods section, these 86 events are manually detected from the tracks of the 96 particles flying over each other. Owing to the fact that our observation is only from one direction, we sometimes can not distinguish clearly whether the apparently overlapping particles are indeed in contact or just in different depths of the view (e.g., those particles on the right side of Fig. 3b). This ambiguity, if happening during the tracking of the particles before and after collisions, casts doubt to the measurement of their velocities and *r*. If we adopt a stricter criterion to exclude those events encountering such ambiguity (shown as the red circles in Fig. 6), the remaining events provide an average value of 〈*r*〉 = 0.982, as indicated by the blue dashed line.

This apparently small difference of 〈*r*〉, however, through the factor of 1/(1 − 〈*r*〉^2^) in Eq. (4), greatly changes *τ*_*k*.*t*._ to the value of about 10 s, now 23% larger than *τ* measured from the cooling. We therefore conclude that the accuracy of our measurement of *r* is limited by our available diagnostics condition. To meet the requirement of such sensitive dependence on *r* and provide a satisfactory quantitative comparison between the theory and the experiment, a precisely calibrated multi-camera system is needed for an unambiguous three-dimensional particle tracking. In addition, the other unaccounted source of energy dissipation, that from the collisions between the particles and the spherical sample cell wall and that from the ambient air drag, also needs three-dimensional analysis and theoretical model to be included in the comparison (see the supplementary material for further discussion).

Figure 6 also shows that we measured *r* > 1 for many collision events, meaning that the translational energy sometimes increases after these energy-dissipating collisions. This can be explained by the conversion of rotational energy into translational degree of freedom. The supplementary video*collision2.avi* records one typical collision event with a strong and visible conversion from rotation to translation, corresponding to an *r* = 1.26, while *collision1.avi* shows one without such conversion, corresponding to an *r* = 0.93.

Villemot et al. shows in theory and simulation^22^ that for prolate ellipsoids with elongation *e* > 1.5, cooling, in the long term, leads to equipartition of energy between the translational and the rotational degrees of freedom. Our particles with *e* = *b*/*a* ≈ 1.9, although indeed in oblate form, shows evidence of energy equipartition as well. As indicated by the black triangles in Fig. 6, the time development of the average *r* value for every 3 s starts above 1 and then decreases below 1 after 6 s into the cooling. This behavior can be interpreted as an initially stronger rotational motion of the particles caused by the astronaut’s manual vibration followed by an overall trend of conversion into translational motion until a balance between the two degrees of freedoms is reached. In this case, however, Eq. (3) becomes invalid since now a time-dependent overall energy input from the rotational degree of freedom can not be addressed by one average value of 〈*r*〉, and a corresponding term needs to be added to the RHS of Eq. (3) (cf.^28^, where the rotational and translational energy are coupled by frictional forces between spherical particles). The cooling time scale from Eq. (4) needs to be changed as well, leading to a different expected value of *τ*_*k*.*t*._. However, due to the lack of rotation measurement, we are not able to quantitatively verify the onset of energy equipartition, as well as how the cooling should behave before this onset. Future improvement in this regard also calls for multi-camera three-dimensional particle tracking setup such as that established in the work by Harth et al.^18^.

In summary, we present in this work a granular gas system of ellipsoidal particles in a long-term low-gravity environment, which enabled us to measure the cooling behavior for a time duration of around 80 s. Two image-processing methods are used to measure the cooling. While the optical flow method shows evidence of a possible clustering behavior, we have to present it with reservation since, again, the two-dimensional image processing may mix clustering with overlapping and may not ascertain if the clustering is caused by the sample cell wall. The trajectory reconstruction and collision detection enabled us to verify the Haff’s cooling of the system as well as to compare the cooling time scale from the experiment with that from the kinetic theory. The lack of accuracy of measuring the *r* value limits us with only a match in the order of magnitude, while the time development of *r* provides interesting evidence of energy equipartition in the rotational and translational degrees of freedom.

## Supplementary information

Supplementary Information

Reporting Summary Checklist

collision1

collision2

## Data Availability

The data and the codes that support the findings of this study are available from the corresponding author upon request.
