# Supplementary material for: Granular cooling of ellipsoidal particles in microgravity
Source: NPJ Microgravity. 2022 Apr 20;8:11. doi: 10.1038/s41526-022-00196-6 (PMC9021203; doi:10.1038/s41526-022-00196-6)
Supplement: Supplementary file 1 — Supplementary Information [file 41526_2022_196_MOESM1_ESM.pdf]

Supplementary Information for 'Granular Cooling of Ellipsoidal Particles in Microgravity'

Sebastian Pitikaris, Patricia Bartz, Peidong Yu[1], Samamtha Christoforetti and Matthias Sperl

Institut für Materialphysik im Weltraum, Deutsches Zentrum für Luft- und Raumfahrt (DLR), 51170 Köln, Germany
Institut für Theoretische Physik, Universität zu Köln, 50937 Köln, Germany
European Astronaut Centre, 51147 Köln, Germany

1 Supplementary Discussion

1.1 Geometric Parameters in Eq. 2

Eq. 2 of the main manuscript uses several geometric parameters defined in our references Villemot & Talbot (2012) and Bereolos *et al.* (1993). We show here some details of their calculation.

Eq. 14 (Villemot & Talbot (2012)) provides the calculation of g_c, which is based on another work (Song & Mason (1990)):

$$\begin{aligned} g_c &= \frac{1-\gamma_1\phi+\gamma_2\phi^2}{(1-\phi)^3}, \\ \gamma_1 &= 3-(1+6\delta+3\delta^2)/(1+3\delta), \\ \gamma_2 &= 3-(1+2.6352\delta+7\delta^2)/(1+3\delta), \\ \delta &= RS/(3V), \end{aligned} \tag{1}$$

where $\phi = 0.036$ is the packing fraction, and $\zeta = \sqrt{1-a^2/b^2}$ and $V = 4\pi ab^2/3$ are the eccentricity and the volume of the ellipsoid respectively. We then follow the oblate parts of Eq. A3 and A4 (Bereolos *et al.* (1993)) to calculate R and S, the surface area and the mean radius of one ellipsoid:

$$\begin{aligned} R &= \frac{a}{2}(1+\frac{b}{a}\cdot\frac{\arcsin(\zeta)}{\zeta}), \\ S &= 2\pi b^2(1+\frac{a^2}{2\zeta b^2}\ln\frac{1+\zeta}{1-\zeta}), \end{aligned} \tag{2}$$

with which, g_c can be eventually calculated using $a = 3.5$ mm and $b = 6.75$ mm of our M&M particles: $g_c = 1.104$.

S_c, the surface area of the excluded volume of two colliding ellipsoids averaged over all relative orientations, is calculated using Eq. 20 (Villemot & Talbot (2012)):

$$S_c = \frac{S}{2\pi} + 2R^2. \tag{3}$$

The result is $S_c = 131.068$ mm^2.

g_c and S_c are analytical integration results of the collisional average of any quantity A defined by Eq. 17 (Villemot & Talbot (2012)). D, the function measuring energy transfer between rotational and translational degrees of freedom, however, must be numerically integrated to obtain $\langle D\rangle_c$. The cumbersome details of this integration calculation can be found in the illustration following Eq. 17 (Villemot & Talbot (2012)). Here we show the results of our numerical integration using python scipy package in Fig. 1. Elongation values (a/b) for both oblate and prolate ellipsoids are used in our numerical integration. The prolate part $(a/b > 1)$ of the curve matches well with the prolate-only result of Villemot & Talbot (2012) shown in their Fig. 11. We then use our oblate parameter $(a/b \approx 0.52)$ to obtain $\langle D\rangle_c = 1.218$.

[1]peidong.yu@dlr.de

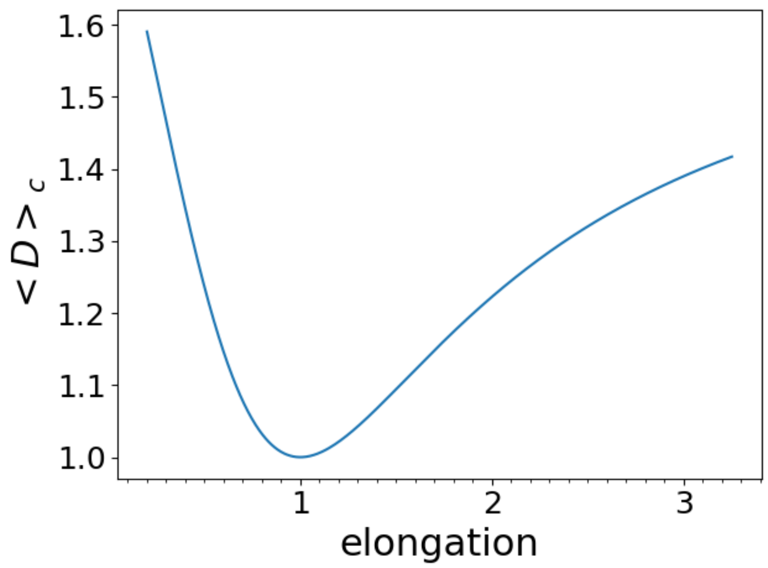

Figure 1: Numerical integration results of $\langle D \rangle_c$ for different elongation values (a/b)

1.2 Mean Free Path of the Particles

We can use the collision rate Γ in Eq. 2 of the manuscript to estimate the mean free path $l \approx v_T/\Gamma(T)$, where $v_T = (\frac{2T}{m})^{\frac{1}{2}}$ is the thermal velocity. Together with Eq. 2 we have $l = (2\sqrt{2\pi} \cdot n g_c S_c \langle D \rangle_c)^{-1} \approx 2.1$ cm. In comparison with the inner diameter of the container 15 cm, we consider that more particle-particle collisions happened than particle-wall collisions, but the energy lost in particle-wall collisions is certainly not negligible. Measurement of this quantity, however, needs three-dimensional imaging.

References

Bereolos, P., Talbot, J., Allen, M. P. & Evans, G. T. 1993 Transport properties of the hard ellipsoid fluid. *J. Chem. Phys.* **99** (8), 6087–6097.

Song, Y. & Mason, E. A. 1990 Equation of state for a fluid of hard convex bodies in any number of dimensions. *Phys. Rev. A* **41** (6), 3121.

Villemot, F. & Talbot, J. 2012 Homogeneous cooling of hard ellipsoids. *Granul. Matter* **14** (2), 91–97.
